# Gallic Acid Can Promote Low-Density Lipoprotein Uptake in HepG2 Cells via Increasing Low-Density Lipoprotein Receptor Accumulation

**DOI:** 10.3390/molecules29091999

**Published:** 2024-04-26

**Authors:** Dongying Zhang, Qixing Zhou, Xiangxuan Yang, Zhen Zhang, Dongxue Wang, Dandan Hu, Yewei Huang, Jun Sheng, Xuanjun Wang

**Affiliations:** 1 College of Science, Yunnan Agricultural University, Kunming 650201, China; zhangdongying365@163.com (D.Z.); hudandan1224@126.com (D.H.); 2 Key Laboratory of Pu-er Tea Science, Ministry of Education, Yunnan Agricultural University, Kunming 650201, China; zhouqixing0515@163.com (Q.Z.); anxuan200@163.com (X.Y.); zhangzhen162897412@163.com (Z.Z.); 15025171365@163.com (D.W.); 3 College of Food Science and Technology, Yunnan Agricultural University, Kunming 650201, China; shengj@ynau.edu.cn; 4 School of Chinese Materia Medica and Yunnan Key Laboratory of Southern Medicinal Resource, Yunnan University of Chinese Medicine, Kunming 650500, China

**Keywords:** gallic acid, NAFLD, LDLR, PCSK9, cholesterol metabolism

## Abstract

Gallic acid (GA) is a type of polyphenolic compound that can be found in a range of fruits, vegetables, and tea. Although it has been confirmed it improves non-alcoholic fatty liver disease (NAFLD), it is still unknown whether GA can improve the occurrence of NAFLD by increasing the low-density lipoprotein receptor (LDLR) accumulation and alleviating cholesterol metabolism disorders. Therefore, the present study explored the effect of GA on LDLR and its mechanism of action. The findings indicated that the increase in LDLR accumulation in HepG2 cells induced by GA was associated with the stimulation of the epidermal growth factor receptor–extracellular regulated protein kinase (EGFR-ERK1/2) signaling pathway. When the pathway was inhibited by EGFR mab cetuximab, it was observed that the activation of the EGFR-ERK1/2 signaling pathway induced by GA was also blocked. At the same time, the accumulation of LDLR protein and the uptake of LDL were also suppressed. Additionally, GA can also promote the accumulation of forkhead box O3 (FOXO3) and suppress the accumulation of hepatocyte nuclear factor-1α (HNF1α), leading to the inhibition of proprotein convertase subtilisin/kexin 9 (PCSK9) mRNA expression and protein accumulation. This ultimately results in increased LDLR protein accumulation and enhanced uptake of LDL in cells. In summary, the present study revealed the potential mechanism of GA’s role in ameliorating NAFLD, with a view of providing a theoretical basis for the dietary supplementation of GA.

## 1. Introduction

In recent years, there has been a worldwide rise in the occurrence of non-alcoholic fatty liver disease (NAFLD), which is now responsible for most instances of long-term liver illnesses [1]. NAFLD has long been considered to be closely related to obesity, dyslipidemia, hypertension, and other diseases. One key factor in the development of NAFLD is the metabolism of cholesterol [2,3]. Cholesterol, which is one of the important components that make up the cell membrane of the body, is a highly hydrophobic lipid and the main raw material for the synthesis of steroid hormones [4]. As the main organ of cholesterol metabolism, the liver maintains the balance of cholesterol metabolism in the liver mainly through biological processes such as endogenous synthesis, uptake, efflux, and esterification. At the same time, the liver is the only organ that can eliminate excess cholesterol by converting it into bile acids and excreting it with bile. However, maintaining the balance of intracellular cholesterol metabolism is an important basis for cells to perform physiological functions [5]. When liver cholesterol metabolism is abnormal, it leads to cholesterol accumulation in the liver to induce NAFLD [6]. Epidemiological studies have suggested a correlation between elevated levels of low-density lipoprotein cholesterol (LDL-C) and a higher prevalence of NAFLD [7]. The primary location of the low-density lipoprotein receptor (LDLR) is on the surface of liver cells, where it plays a role in regulating blood lipid levels and maintaining fibrinolytic function. LDLR is responsible for clearing and metabolizing over 70% of LDL-C in the liver [8]. Therefore, LDLR is critical in aiding LDL-C elimination. Additionally, enhancing the level of LDLR accumulation is a key approach for managing and preventing NAFLD.

The regulation of LDLR levels is a complex multi-layered regulatory mechanism involving the transcriptional level, the post-transcriptional level, and the post-translational level. At the transcriptional level, LDLR mRNA expression is controlled by a negative feedback mechanism of cholesterol reactivity, which is achieved by sterol-responsive element binding protein 2 (SREBP2) [9]. At the post-transcriptional level, LDLR mRNA stability is one of the key factors in its regulation. For example, berberine (BBR) enhances LDLR mRNA stability by activating the ERK pathway, which in turn enhances LDLR protein accumulation [10,11]. The main control of LDLR’s post-translational regulation comes from PCSK9, which leads to its degradation by binding to LDLR [12]. Prior research has indicated that the LDLR mRNA has a half-life of around 30 min [13]. Activation of the signaling pathway involving the epidermal growth factor receptor–extracellular regulated kinase (EGFR-ERK1/2) can extend the lifespan of LDLR mRNA, leading to an increase in the accumulation level of LDLR protein [14,15,16]. Based on the information provided, it has been reported that berberine, a type of alkaloid compound categorized within the isoquinoline sesquiterpene group, is capable of enhancing LDLR accumulation through the activation of the ERK1/2 signaling pathway [17,18]. This activation can help improve NAFLD caused by disruptions in cholesterol metabolism. Meanwhile, there is increasing evidence indicating that the proprotein convertase subtilisin/kexin 9 (PCSK9) is associated not only with autosomal dominant hypercholesterolemia but also has a substantial impact on the regulation of lipid metabolism in the body [19]. PCSK9 regulates hepatic LDLR by binding to the cell surface of LDLR after it has been translated. The PCSK9 protein secreted out of hepatocytes binds to LDLR, thereby forming a PCSK9-LDLR complex and inducing the degradation of LDLR in lysosomes, reducing the recycling of LDLR [20,21,22]. PCSK9 is controlled by a variety of transcriptional regulators at the transcriptional level. In liver cells, hepatocyte nuclear 1α (HNF1α) can bind to the PCSK9 promoter and promote the accumulation of PCSK9 [23]. In contrast, the forkhead box O3a (FoxO3) protein interacts with the PCSK9 promoter, recruits sirtuin6 to the proximal promoter region of the PCSK9 gene, acetylates histone H3, and inhibits PCSK9 transcriptional accumulation [24]. The production of PCSK9 is also controlled by SREBP2 through transcriptional regulation. When cellular cholesterol levels are depleted, activated SREBP-2 moves into the nucleus, binds to the PCSK9 promoter (SRE-1 region), and promotes enhanced transcription and translation of PCSK9 [25].

Gallic acid (GA), also called 3,4,5-trihydroxybenzoic acid, is a type of polyphenolic compound found in vegetables, fruits, red wine, and tea as it is part of hydrolysable tannins [26,27]. The health benefits and high quality of tea are well known, as it is made from the young leaves of the tea tree. Studies have indicated that the amount of GA in various types of tea can vary from 0.1% to 2% [28]. Related findings suggest that GA can ameliorate metabolic diseases such as diabetes and NAFLD by reducing blood glucose and hepatic lipid peroxidation through the upregulation of peroxisome proliferator-activated receptor (PPAR) in liver, muscle, and adipose tissue [27,29,30]. In addition, cell experiments confirmed that GA can reduce cholesterol levels in 3T3-L1 adipocytes [31]. Furthermore, GA can reduce lipid and cholesterol accumulation in HepG2 cells by inhibiting the activity of enzymes related to fatty acid and cholesterol synthesis [32]. Although these findings confirm that GA improvement has a role in NAFLD. However, the mechanism by which GA slows down the occurrence of NAFLD by alleviating cholesterol metabolism disorders has not yet been revealed [33]. As a result, the current research investigated how GA affects the regulation of LDL-C metabolism and its molecular mechanism in HepG2 cells by enhancing LDL uptake through the increased accumulation of LDLR.

## 2. Results

### 2.1. The Accumulation of LDLR Was Increased by GA, Leading to an Enhancement in the Uptake of LDL in HepG2 Cells

**Figure 1:**
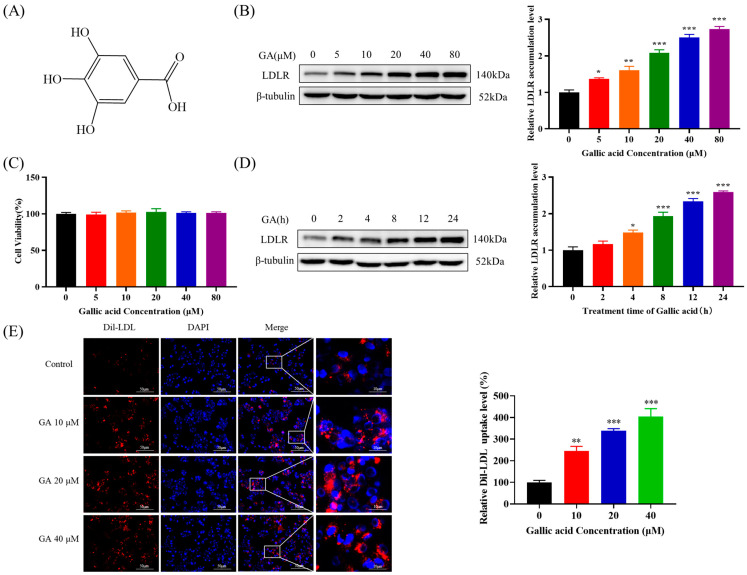
The administration of GA resulted in an upregulation of LDLR accumulation and enhanced LDL uptake in HepG2 cells: (**A**) GA molecular structure; (**B**,**D**) HepG2 cells were subjected to GA treatment at various concentrations and durations, followed by quantification of LDLR protein levels via Western Blot analysis; (**C**) HepG2 cells were exposed to various concentrations of GA (5, 10, 20, 40, and 80 μM) for a duration of 24 h, and the assessment of cell viability was conducted using the MTT assay; and (**E**) HepG2 cells were exposed to different concentrations of GA (10, 20, and 40 μM) for a duration of 20 h. Subsequently, the cells were treated with Dil-LDL (20 μg/mL) for a period of 4 h. The uptake activity of LDL was then visualized using fluorescence microscopy. Values are presented as means ± SEM (n = 3). *: *p* < 0.05, **: *p* < 0.01, ***: *p* < 0.001 compared with control group.

Firstly, the effect of GA on HepG2 cell viability was examined in this study using the MTT assay, and the results indicated that the viability and proliferation of HepG2 cells were not significantly affected after being exposed to various concentrations of GA (0, 5, 10, 20, 40, and 80 μM) for a duration of 24 h (Figure 1C). Subsequent experiments were performed in this concentration range. Moreover, the study examined the effect of GA treatment on the expression of LDLR protein in HepG2 cells. The results suggested that GA was able to enhance the accumulation of LDLR protein in HepG2 cells in a manner dependent on both concentration and time (Figure 1B,D). Additionally, to validate the impact of GA on the uptake of LDL in HepG2 cells, 1,1′-dioctadecyl-3,3,3′,3′-tetramethylindocarbocyanide perchlorate (Dil)–LDL was employed for the purpose of tagging LDL within cells. The findings indicated that the uptake of LDL by HepG2 cells was notably enhanced following GA treatment in comparison to the control group (Figure 1E). These results indicated that GA could potentially enhance the uptake of LDL in HepG2 cells by upregulating the production of LDLR protein.

### 2.2. The EGFR-ERK1/2 Signaling Pathway Was Activated by GA, Leading to an Enhancement of LDLR Accumulation in HepG2 Cells

**Figure 2:**
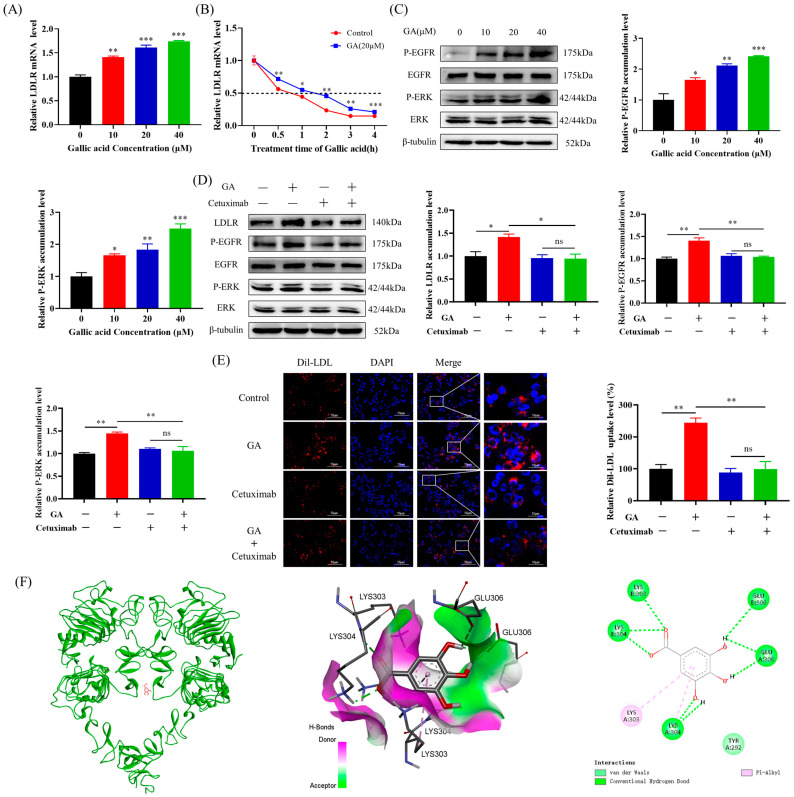
The EGFR-ERK1/2 signaling pathway was activated by GA, leading to an increase in LDLR accumulation and improved uptake of LDL in HepG2 cells: (**A**) The HepG2 cell line was exposed to gallic acid at concentrations of 10, 20, and 40 μM for a duration of 24 h. The LDLR mRNA levels were measured by RT-PCR. (**B**) The HepG2 cells were exposed to actinomycin D (Act D: 5 µg/mL) for 30 min, and then treated with or without 20 μM GA at intervals of 0.5 h, 1 h, 2 h, 3 h, and 4 h for RNA extraction. The impact of GA on LDLR mRNA half-life was evaluated using RT-qPCR. (**C**) The HepG2 cell line was treated with varying concentrations of GA (10, 20, and 40 μM) for a duration of 24 h. Subsequently, the effect of GA on the levels of p-EGFR, EGFR, p-ERK1/2, and ERK1/2 proteins was evaluated through Western Blot analysis. (**D**) The EGFR signaling pathway in HepG2 cells was blocked by treating them with cetuximab for 1 h, followed by a 24 h treatment with GA (20 μM). Western Blot analysis was performed to evaluate the effects of 20 μM GA treatment on LDLR, p-EGFR, EGFR, p-ERK1/2, and ERK1/2 protein levels in HepG2 cells. (**E**) The HepG2 cells were first exposed to cetuximab for 1 h to block the EGFR signaling pathway. This was followed by treatment with GA (20 μM) for 20 h and then Dil-LDL (20 μg/mL) for 4 h. The effect of GA on the LDL uptake activity of the inhibited EGFR signaling pathway was then observed using inverted fluorescence microscopy. (**F**) 3D schematic diagram (EGFR: green; GA: red), hydrogen bond receptor surface schematic diagram, and 2D schematic diagram of docking between gallic acid and EGFR extracellular domain. Values are presented as means ± SEM (n = 3). *: *p* < 0.05, **: *p* < 0.01, ***: *p* < 0.001 compared with control group.

The above results suggest that GA is able to enhance LDL uptake by promoting LDLR protein accumulation, and LDLR protein levels are generally regulated by two pathways: one approach is to enhance the production of LDLR mRNA, while the other is to prevent the breakdown of LDLR by PCSK9. Therefore, the present study explored the effect of GA on LDLR mRNA stability. The present study investigated the impact of GA on LDLR mRNA expression and demonstrated that GA significantly increased the expression of LDLR mRNA in HepG2 cells (Figure 2A). Then, this research employed Actinomycin D to assess the impact of GA on the stability of LDLR mRNA. The results suggested that GA can significantly extend the half-life of LDLR mRNA, increasing the half-life of LDLR mRNA from approximately 0.59 h to approximately 1.02 h, thereby improving the stability of LDLR mRNA (Figure 2B).

According to relevant research, an overactive EGFR-ERK1/2 signaling pathway has been found to inhibit the degradation of LDLR mRNA and enhance the LDLR accumulation [16]. It was observed that treatment with GA led to a significant increase in the levels of phosphorylated EGFR and ERK1/2 compared to those of the control group (Figure 2C). For reconfirming the association between GA’s promotion of LDLR accumulation and activation of the EGFR-ERK1/2 signaling pathway, additional experiments were conducted in this study to assess the impact of GA on LDLR accumulation and LDL uptake in HepG2 cells when cetuximab is used to block the EGFR signaling pathway. The results suggested that cetuximab could counteract the stimulatory effect of GA on the pathway, as well as hinder the rise in LDLR protein level and uptake of LDL caused by GA (Figure 2D,E).

Molecular docking can effectively predict the drug binding mode and the molecular mechanism. To further investigate whether GA can specifically bind to the extracellular segment of EGFR to alter its conformation and activate its phosphorylation level, molecular docking between GA (CAS: 149-91-7) and EGFR (PDB ID: 3njp) was performed using AutoDock 4.2.6. The binding mode of GA in the active pocket of EGFR is shown in Figure 2F. GA is able to form hydrogen bonds with LYS304, LYS303, and GLU306 of EGFR, indicating that hydrogen bonds play an important role in the interaction between GA and EGFR. Moreover, there is a hydrophobic interaction between GA and TYR292. Further calculation of the affinity between GA and EGFR revealed that the binding energy between the two was −6.97 kcal/mol. It is generally accepted that components with binding energy of <0 kcal/mol can spontaneously bond. The lower the binding energy value, the more stable the binding conformation and the greater the interaction between the ligand and the receptor. Therefore, we hypothesized that GA binds specifically to the active pocket of EGFR by forming hydrogen bonds (LYS304, LYS303, and GLU306) and hydrophobic interactions (TYR292) with specific amino acids. This binding not only enhances the interaction force between GA and EGFR but also helps to maintain the structural stability of the EGFR-GA complex, thereby changing the conformation of EGFR. The conformational change in EGFR activates the EGFR-ERK signaling pathway by promoting its phosphorylation protein level.

The results suggested that GA might have a direct impact on the extracellular region of EGFR, leading to the self-phosphorylation of EGFR and the activation of the downstream ERK1/2 signaling pathway. This ultimately resulted in a prolonged LDLR mRNA half-life, promoting increased LDLR accumulation and LDL uptake.

### 2.3. The Accumulation of PCSK9 Was Inhibited by GA, Leading to an Enhancement in Accumulation of LDLR in HepG2 Cells

**Figure 3:**
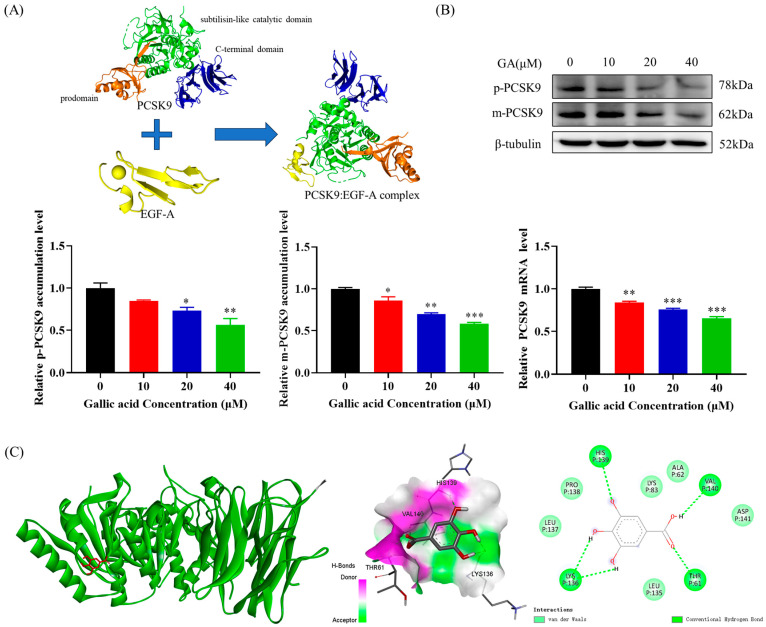
Repression of PCSK9 accumulation and its binding with LDLR in HepG2 cells is inhibited by GA: (**A**) The catalytic structural domain of PCSK9 (green) and the EGF-A region of LDLR (yellow) both bind to form the PCSK9: EGF-A complex. (**B**) The HepG2 cell line was treated with GA at 10, 20, and 40 μM concentrations for a period of 24 h. After the treatment, the levels of PCSK9 mRNA and protein were analyzed using Western Blot and RT-PCR. (**C**) 3D schematic diagram (PCSK9: green; GA: red), hydrogen bond receptor surface schematic diagram, and 2D schematic diagram of GA docking with PCSK9. Values are presented as means ± SEM (n = 3). *: *p* < 0.05, **: *p* < 0.01, ***: *p* < 0.001 compared with control group.

PCSK9 interactes with the terminal extracellular domain of LDLR and guides LDLR degradation (Figure 3A). Hence, it is theoretically possible to manipulate the post-transcriptional accumulation of PCSK9 either directly or indirectly, with the aim of reducing the interaction between PCSK9 and LDLR in order to lessen lysosomal-mediated degradation of LDLR. This research delved deeper into the influence of GA on PCSK9 accumulation and explored the potential correlation between GA and PCSK9, which indicated that GA inhibited PCSK9 mRNA expression and protein accumulation in a concentration-dependent manner (Figure 3B). The binding mode of GA (CAS: 149-91-7) specifically bound to the active pocket position of PCSK9 (PDB ID:2P4E) was predicted using molecular docking. The results showed a good conformational match of GA at the position of PCSK9 active pocket (Figure 3C), which indicated that GA was able to bind to the active pocket region of PCSK9 in a stable manner. This stability was mainly due to the hydrogen bonds between GA and the amino acid residues HIS139, VAL140, THR61, and LYS136 of PCSK9, as well as the hydrophobic interactions with LEU137, PRO138, LYS83, ALA62, ASP141, and LUE135. The binding energy was further found to be −5.36 kcal/mol. These interactions not only strengthen the binding affinity between GA and PCSK9 but also facilitate GA’s specific binding to the “active pocket” of PCSK9 and induce a change in the conformation of PCSK9. These findings suggest that GA can specifically bind to the active pocket of PCSK9 through stable hydrogen bonds and hydrophobic interactions and may indirectly impact LDL-C metabolism by modulating the conformation of PCSK9 to inhibit its activity or enhance LDLR activity. In conclusion, GA inhibits the activity of PCSK9 and may directly act on PCSK9 to inhibit the binding of PCSK9 to LDLR, thereby reducing the degradation of LDLR.

### 2.4. GA Activated FOXO3 Accumulation and Inhibited HNF1α and SREBP2 Accumulation in HepG2 Cells

**Figure 4:**
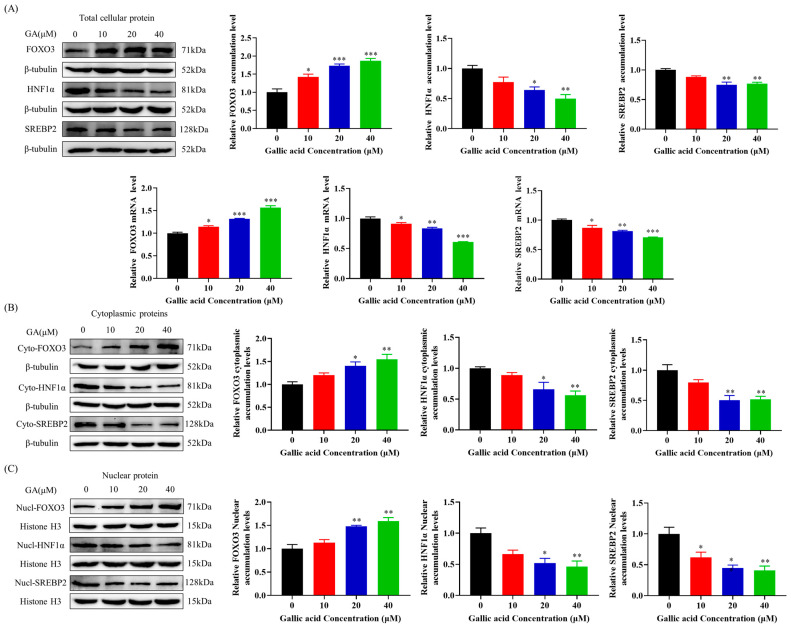
GA decreased PCSK9 accumulation in HepG2 cells by increasing FOXO3 accumulation and inhibiting HNF1α and SREBP2 accumulation: (**A**) HepG2 cells were exposed to GA (10, 20, and 40 μM) for a duration of 24 h, as well as FOXO3, HNF1α, and SREBP2 mRNA and protein levels were measured by Western Blot and RT-PCR and (**B**,**C**) GA (10, 20, 40 μM) was used to treat HepG2 cells for 24 h, and FOXO3, HNF1α, and SREBP2 cytosis and nuclear protein levels were measured through Western Blot. Values are presented as means ± SEM (n = 3). *: *p* < 0.05, **: *p* < 0.01, ***: *p* < 0.001 compared with control group.

From the above results, it can be seen that GA reduced LDLR degradation by inhibiting the accumulation of PCSK9. The transcriptional regulation of PCSK9 involves the control of its expression by a range of factors, one of which is SREBP2. In order to delve deeper into the molecular mechanism behind the decrease in PCSK9 mRNA expression and protein accumulation induced by GA, this research conducted a quantitative analysis of the important transcription factors FoxO3, HNF1α, and SREBP2 of PCSK9. The results showed that GA significantly increased FOXO3 levels while decreasing HNF1α and SREBP2 levels, both at the protein accumulation and transcription levels (Figure 4A). FoxO3, HNF1α, and SREBP2, as nuclear transcription factors, were transferred to the nucleus to exert transcriptional activity. As a result, this research delved deeper into the effects of GA on the protein concentrations of FoxO3, HNF1α, and SREBP2 in both the cytoplasmic and nuclear compartments of HepG2 cells. The findings indicated that GA resulted in a notable increase in FOXO3 protein levels, while leading to a reduction in HNF1α and SREBP2 protein levels in both the cytoplasm and the nucleus (Figure 4B,C). Although this study has proved that GA can inhibit the accumulation of SREBP2, there are still some shortcomings in this study. SREBP2 is a transcription factor located on the endoplasmic reticulum membrane, and its activity is regulated by cholesterol levels. When intracellular cholesterol levels rise, SREBP cleavage-activating protein (SCAP) binds to SREBP2 and is transported to the Golgi for processing. Finally, SREBP2 is released into the nucleus to activate the expression of related genes and promote the synthesis of cholesterol and fatty acids [34]. When cholesterol levels in hepatocytes are reduced (for example, cholesterol levels in blood are decreased due to increased LDLR), Insulin-Induced Gene 1/2 (INSIG1/2) and SCAP binding prevents SREBP2 transport to the Golgi for processing, thereby inhibiting SREBP2 accumulation [35]. Therefore, it remains to be further investigated whether the decrease in SREBP2 accumulation is due to the effect of GA or an increase in LDLR accumulation. These results suggest that GA was able to reduce PCSK9 accumulation by increasing FOXO3 accumulation while inhibiting HNF1α accumulation.

## 3. Discussion

Nonalcohol fatty liver disease (NAFLD) is a major health problem leading to hepatic and extrahepatic morbidity, with a global prevalence as high as 25.2% [36,37]. With the emergence of new research results, the traditional “double whammy” theory can no longer fully explain the pathogenesis of NAFLD. Currently, NAFLD is generally considered to be associated with risk factors for metabolic diseases such as obesity, dyslipidemia, hypertension, and diabetes [38]. Cardiovascular disease (CVD), the leading worldwide reason for death, is the primary cause of mortality in NAFLD patients, particularly Atherosclerosis (AS) [39]. Low-density lipoprotein cholesterol (LDL-C) is derived from Very LDL-C (VLDL-C) in the circulatory system, predominantly produced in the blood vessels and primarily metabolized in the liver. Elevated levels of LDL-C in the liver have been linked to the development of non-alcoholic fatty liver disease [7]. Recent studies on the spread of diseases have suggested that higher levels of LDL-C may potentially increase the risk of developing non-alcoholic fatty liver disease [40]. In addition, the potential beneficial effects of ezetimibe and atorvastatin, which are commonly used clinically for the treatment of hyperlipidemia, may also be mediated by a reduction in hepatic cholesterol levels [41,42]. Recent research has shown that the low-density lipoprotein receptor (LDLR) accumulation in the liver is essential. The clearance of low-density lipoprotein (LDL) in the bloodstream by hepatocyte entosis mediated by LDLR, as well as the feedback regulation of endogenous cholesterol synthesis, is a crucial regulatory mechanism for maintaining optimal levels of LDL in the blood [43]. Therefore, the management of NAFLD induced by elevated cholesterol levels may include upregulating LDLR accumulation or suppressing proprotein convertase subtilisin/kexin9 (PCSK9)-mediated LDLR degradation to enhance LDLR levels.

Plant polyphenols, also known as plant tannins, are a class of secondary metabolites that are widely present in plants with complex chemical structures and a wide range of pharmacological properties. Plant polyphenols have become an increasingly important area of research in food science due to their diverse physiological effects. As a naturally abundant endogenous plant phenol, gallic acid (GA) can improve NAFLD by increasing the antioxidant capacity of the liver and inhibiting the ROS/NF-κβ/TNFα inflammatory pathway [44,45]. Moreover, GA can also lower cholesterol levels in 3T3-L1 adipocytes and decrease lipid and cholesterol accumulation in HepG2 cells by inhibiting the activities of enzymes involved in fatty acid and cholesterol synthesis [31,32]. These results indicated that GA could improve NAFLD. However, the effect of GA on LDLR has not been reported. LDLR is a key protein that regulates cholesterol levels in the blood, and its dysfunction is closely related to the development of NAFLD. In this study, it was found that GA induced a significant increase in LDLR protein level in HepG2 cells in a concentration and time-dependent manner (Figure 1B,D). In addition, the uptake of LDL by HepG2 cells was significantly promoted after GA treatment compared with the control group (Figure 1E). Although the present study demonstrated that GA could promote the uptake of LDL by HepG2 cells by increasing LDLR activity by Western Blot and 1,1′-dioctadecyl-3,3,3′,3′-tetramethylindocarbocyanide perchlorate (Dil)–LDL uptake assay, related studies have shown that HepG2 cells can increase intracellular cholesterol levels by synthesis or uptake when the intracellular free cholesterol level is reduced. The excess intracellular free cholesterol is balanced by efflux and esterification [46]. Therefore, further studies are necessary to investigate the effect of GA on free cholesterol and esterified cholesterol levels in HepG2 cells.

Further studies showed that the accumulation of LDLR protein in HepG2 cells induced by GA was related to the activation of epidermal growth factor receptor–extracellular regulated protein kinase (EGFR-ERK1/2) signaling pathway. This study demonstrated that GA was able to improve LDLR mRNA stability and promote LDLR accumulation by activating the EGFR-ERK1/2 pathway in HepG2 cells, and this effect was inhibited when EGFR signaling was blocked by cetuximab (Figure 2A–E). In other words, GA activated the EGFR-ERK1/2 signaling pathway to enhance LDLR mRNA stability and induced LDLR mRNA expression and protein accumulation. It has been reported that the post-transcriptional regulation of LDLR is mainly achieved by regulating the stability of its mRNA [10]. LDLR mRNA is unstable, and its rapid degradation is controlled by a Au-enriched element (ARE1) present in the 3′-untranslated region (3′-UTR) of LDLR mRNA. The ARE-binding protein (ARE-BP) binds specifically to ARE1, thereby promoting LDLR mRNA instability, an effect that is inhibited when ERK1/2 is activated [13,47]. Relevant studies have shown that some natural small molecules such as berberine and ellagaloic acid can stabilize LDLR mRNA levels through the ERK1/2 pathway, thereby improving NAFLD [48,49]. However, unlike berberine and ellagic acid, the molecular docking results in this study showed that GA was able to bind to the extracellular domain of EGFR, forming a strong affinity through hydrogen bonding and hydrophobic interaction (Figure 2F). These interactions enable GA to specifically bind to the extracellular domain of EGFR and induce the conformational change in EGFR, which ultimately leads to the activation of EGFR-ERK1/2 signaling pathway. Although this study confirmed that GA can enhance LDLR mRNA stability at the post-transcriptional level by activating EGFR-ERK1/2 signaling pathway, the exact mechanism of post-transcriptional regulation and the functional proteins involved in the process of enhancing LDLR mRNA stability need to be further investigated. This study suggested that GA may reduce the binding of ARE-BP to the 3′-UTR of the unstable LDLR mRNA, thereby promoting its stabilization and increasing LDLR accumulation [47]. In addition, the application of small interfering RNA (siRNA) technology in this study to further demonstrate the effect of GA through EGFR-ERK1/2 pathway needs to be further studied. As a kind of double-stranded short RNA molecule composed of 21–25 nucleotides, siRNA can specifically induce the silencing of target genes, and then inhibit the accumulation of proteins. This technology is based on the RNA interference (RNAi) mechanism, in which the expression of specific genes is silenced by the recognition and binding of specific siRNA molecules to the target mRNA, resulting in degradation or translation block of the target mRNA [50]. Therefore, transfection of EGFR and ERK1/2 siRNA into HepG2 cells can not only further inhibit the accumulation of EGFR and ERK proteins but also provide another effective research method for studying the effect of GA on the EGFR-ERK1/2 signaling pathway.

Additionally, this study also found that GA could inhibit PCSK9 mRNA expression and protein accumulation in HepG2 cells, which in turn increased LDLR protein accumulation and increased LDLR uptake (Figure 3B). PCSK9, a serine protease produced by hepatocytes, serves as a crucial modulator of LDLR and exerts significant influence on the regulation of plasma LDL-C levels. A research has shown that the catalytic domain of PCSK9 binds to the extracellular epidermal growth factor-like repeat A (EGF-A) domain of LDLR (Figure 3A), and this binding leads to the transfer of LDLR to lysosomes for degradation, resulting in a decrease in LDLR on the hepatocyte surface and an increase in plasma levels of LDL-C [51]. It was further found that GA could inhibit the accumulation of PCSK9 by activating forkhead box O3 (FOXO3) and inhibiting the accumulation of hepatocyte nuclear factor-1α (HNF1α), which in turn favored the accumulation of LDLR protein and the uptake of LDL (Figure 4A–C). In liver tissue, the transcriptional level of PCSK9 is mainly regulated by HNF1α, FoxO3, sterol-responsive element binding protein 2 (SREBP2), and other transcription factors. Related studies have shown that HNF1α binds to the HNF1 motif located upstream of SRE1 in the PCSK9 promoter to promote PCSK9 transcription, whereas FoxO3 interacts with the PCSK9 promoter and inhibits PCSK9 transcription expression [52,53]. Meanwhile, mutations in the HNF1α binding site significantly reduced the SREBP2-mediated upregulation of PCSK9 transcription. Thus, HNF1α is able to cooperate with SREBP2 to activate PCSK9 transcription [23]. It is worth noting that, although this study found that GA could inhibit the accumulation of SREBP2 (Figure 4A–C), it cannot be denied that this study could not prove the specific role of SREBP2 in the regulation of LDLR. When cholesterol levels in hepatocytes are reduced (for example, cholesterol levels in blood are decreased due to increased LDLR), Insulin-Induced Gene ½ (INSIG1/2) and SREBP cleavage-activating protein (SCAP) binding prevents SREBP2 transport to the Golgi for processing, thereby inhibiting the SREBP2 activity [35]. Molecular docking results showed that GA could specifically bind to the active pocket of PCSK9 and cause a conformational change to inhibit the binding of PCSK9 to LDLR, thereby reducing the degradation of LDLR (Figure 3C).

## 4. Materials and Methods

### 4.1. Materials and Reagents

Gallic acid (GA) (HPLC ≥ 98%) from Shanghai yuanye Bio-Technology Co., Ltd., Shanghai, China; Trypsin–EDTA (0.25%), penicillin–streptomycin, phenylmethanesulfonyl fluoride (PMSF), 3-(4,5-dimethylthiazol-2-yl)-2,5-diphenyltetrazolium bromide (MTT), and Trizol reagent from Thermo Fisher Scientific, Shanghai, China; Dulbecco’s modified Eagle’s medium (DMEM) and Fetal Bovine Serum (FBS) from MeilunBio; 4′,6-diamidino-2-phenylindole (DAPI) and Human 1,1′-dioctadecyl-3,3,3′,3′-tetramethylindocarbocyanide perchlorate–low-density lipoprotein (Human Dil-LDL) from Yeasen Biotechnology (Shanghai) Co., Ltd., Shanghai, China; SYBR Green real-time PCR Master Mix from Takara Biomedical Technology (Beijing) Co., Ltd., Beijing, China; Kit for the nuclear extraction of the cell from Beijing Solarbio Science & Technology Co., Ltd., Beijing, China; BCA Protein Reagent from Shanghai Beyotime Biotechnology Co., Ltd., Shanghai, China; anti-LDLR, ERK1/2, phospho-ERK1/2, EGFR, phospho-EGFR, and β-tubulin from HuaBio, Hangzhou, China; and Anti-PCSK9, HNF1α, FOXO3, and SREBP-2 from Wuhan ABclonal Biotechnology Co., Ltd., Wuhan, China.

### 4.2. Cell Culture

Upon reaching a cell density of 80–90%, well-developed hepatocellular carcinoma HepG2 cells (from the Kunming Cell Bank of the Chinese Academy of Sciences) are introduced into high-glucose DMEM medium with 1% penicillin–streptomycin and 10% FBS at a concentration of 1.5 × 10^5^/mL and are then incubated in a constant temperature environment (37 °C, 5% CO_2_).

### 4.3. MTT Assay

The HepG2 cells were plated in 96-well plates at a concentration of 2 × 10^4^ cells/well, and the outer wells were filled with sterile PBS. They were then incubated in a 5% CO_2_ and 37 °C environment for 12–24 h until reaching the desired cell density. After that, GA (0, 5, 10, 20, 40, or 80 μM) was added under light-free conditions and incubated for an additional period of 24 h. Next, 20 μL of a solution containing 5% MTT was added to each well, and the plate was placed in a dark environment for 4–5 h with aluminum foil covering. Following the incubation period, the culture was terminated, and the liquid from each well was carefully removed. Next, 200 µL of dimethyl sulfoxide (DMSO) was introduced into each well and gently agitated on a low-speed shaker for 15 min to facilitate the dissolution of the formazan crystals. The absorbance readings for each well were then taken at 492 nm using a multi-purpose enzyme reader.

### 4.4. Western Blot Analysis

First, various concentrations of GA (0, 10, 20, and 40 μM) were administered to HepG2 cells for a duration of 24 h, and protease and phosphatase inhibitors were added to RIPA buffer to extract the total protein of HepG2 cells. The cell extract underwent centrifugation at 1000–15,000× *g* for 15 min at 4 °C. The resulting supernatant was utilized for total protein analysis, with cytoplasmic and nuclear proteins extracted from the cells following the guidelines of the nuclear protein extraction kit. The BCA protein assay reagent was utilized to determine the overall protein concentration. A total of 50 μg of protein was then evenly distributed and transferred onto a 0.45 μm PVDF membrane using 8% SDS-PAGE. After being blocked in TBST with 5% bovine serum albumin for 1 h at room temperature, the membrane was subsequently incubated with rabbit anti-LDLR antibody overnight at 4 °C.

### 4.5. Dil-LDL Uptake Test

The HepG2 cells were exposed to varying levels of GA (0, 10, 20, and 40 μM) for a period of 20 h. The DiI-LDL was diluted to a concentration of 30 µg/mL in the cell medium under sterile and dark conditions. Subsequently, the medium from the culture plate was removed in the absence of light, and the aforementioned LDL was introduced to the live cells for incubation at 37 °C for a period of 4 h. After the cells were cultured, the solution with Human DiI-Ox-LDL was removed and rinsed three times with PBS. The cells were then fixed in 3% formaldehyde at room temperature for a duration of 20 min and counterstained with DAPI-containing anti-fade mounting solution for nuclear restaining. The cells were ultimately observed using fluorescence microscopy with an inverted configuration (Ex/Em = 549 nm/565 nm) to assess the LDLR uptake activity on the membrane surface. Subsequently, the quantitative analysis of immunofluorescence was conducted utilizing Image J.

### 4.6. Quantitative Reverse Transcription Polymerase Chain Reaction (RT-qPCR)

First, HepG2 cells were treated with different concentrations of GA (0, 10, 20, and 40 μM) for 24 h. Following the cell treatment, the cells were rinsed three times with PBS after removing the medium. Then, 1 mL of Trizol reagent was used to lyse HepG2 cells for total RNA extraction according to the manufacturer’s instructions. Reversing the instructions provided in the kit, cDNA was synthesized from 1 μg of total RNA, and real-time quantitative PCR was performed on the LightCycler480 system with SYBR Green as the template. The relative expression levels of relevant mRNA were measured after normalization with β-actin levels using the 2^−ΔΔCt^ method. The detailed primer sequence for quantitative real-time PCR analysis can be found in Table 1.

### 4.7. Molecular Docking

Molecular docking of GA with EGFR and PCSK9 was performed using AutoDock4.2.6. Firstly, download the EGFR (PDB ID: 3njp) and PCSK9 (PDB ID: 2P4E) protein structures from the PDB database (https://www.rcsb.org/, accessed on 14 December 2023) and import them into PyMOL2.5 to remove excess ligands. Secondly, AutoDock4.2.6 was used to preprocess the structures of EGFR and PCSK9 protein molecules by dehydration, hydrogenation, etc. and use them as receptors. Then, the 3D structure of GA (CAS: 149-91-7) in SDF format was downloaded from the PubChem database (https://pubchem.ncbi.nlm.nih.gov/, accessed on 14 December 2023), imported into Chem3D 20.0 for energy and structure optimization, and the Minimum RMS Gradient was set to 0.001, and it was used as a ligand. Finally, molecular docking was performed, and binding energies were calculated using AutoDock 4.2.6, and graphics were created using Discovery Studio 2021.

### 4.8. Data Analysis and Mapping

The experimental data were assessed using GraphPad Prism 9.0 to establish significance and for the purpose of visualization. Statistical analysis utilized the Student t test, and the findings are displayed as the mean ± standard error (SEM) derived from a minimum of three separate experiments (*: *p* < 0.05, **: *p* < 0.01, ***: *p* < 0.001).

## 5. Conclusions

**Figure 5:**
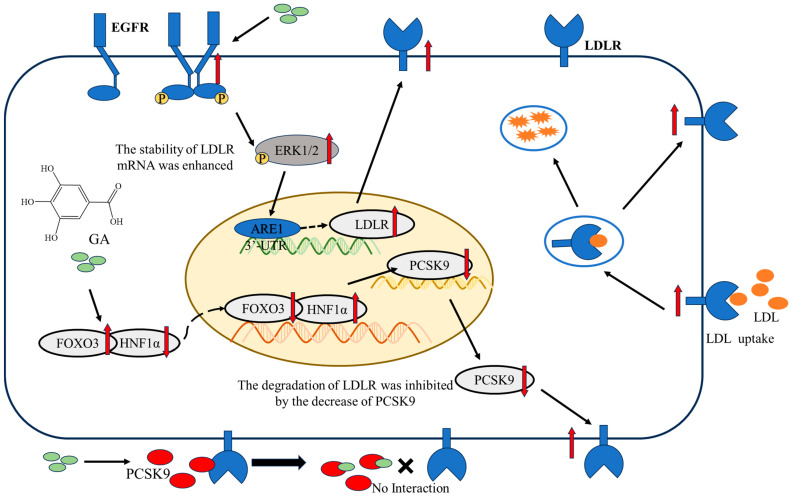
Schematic representation of the pathway mechanism by which GA can promote LDL uptake by HepG2 cells by increasing LDLR accumulation.

In summary, GA had the ability to stimulate the EGFR-ERK1/2 signaling pathway by specifically focusing on the EGFR extracellular domain. This leads to a longer lifespan of LDLR mRNA and improved stability, ultimately leading to an increase in LDLR accumulation and an enhanced uptake of LDL in HepG2 cells. Moreover, GA can also reduce the expression of PCSK9 mRNA and the accumulation of protein by activating FOXO3 and inhibiting the accumulation of HNF1α. Simultaneously, GA may act directly on PCSK9 to block its binding to LDLR, which in turn reduces the degradation of LDLR protein, thereby increasing the uptake of LDL (Figure 5). Overall, this research has clarified how GA works to increase LDLR accumulation and LDL uptake in HepG2 cells, laying a new theoretical foundation for the development of NAFLD drugs. These significant findings will also provide fresh perspectives on exploring natural remedies for preventing and treating NAFLD, effectively utilizing the advantages and characteristics of natural products in managing the condition.

## Figures and Tables

**Table 1:** Primer sequences used for quantitative real-time PCR analysis in this study.

Genes	Primer Sequences (5′-3′)	
LDLR	F: GAACCCATCAAAGAGTGCG	R: TCTTCCTGACCTCGTGCC
PCSK9	F: CCAAGCCTCTTCTTACTTCACC	R: GCATCGTTCTGCCATCACT
SREBP2	F: CCCTGGGAGACATCGACGA	R: CGTTGCACTGAAGGGTCCA
HNF1α	F: GTGGCGAAGATGGTCAAGTCC	R: CCCTTGTTGAGGTGTTGGG
FOXO3	F: ACATGGGCTTGAGTGAGT	R: GCCTGAGAGAGAGTCCGAGA
β-actin	F: ACAGAGCCTCGCCTTTGCCG	R: ACATGCCGGAGCCGTTGTCG

## Data Availability

Data will be made available on request.
